# Supplementary material for: Excited-State Forces with GW-BSE Through the Hellmann–Feynman Theorem
Source: Int J Mol Sci. 2025 Mar 5;26(5):2306. doi: 10.3390/ijms26052306 (PMC11900226; doi:10.3390/ijms26052306)
Supplement: Supplementary file 1 [file ijms-26-02306-s001.zip › ijms-3417018-supplementary.pdf]

Supporting Information for: Excited states forces with GW-BSE through the Hellmann-Feynman theorem

Marah Jamil Alrahamneh,[†] Iogann Tolbatov,[†] and Paolo Umari[*,†,‡]

†*Dipartimento di Fisica e Astronomia, Università di Padova, I-35131 Padova, Italy*

‡*CNR-IOM DEMOCRITOS, Istituto Officina dei Materiali, Consiglio Nazionale delle Ricerche, I-34149 Trieste, Italy*

E-mail: paolo.umari@unipd.it

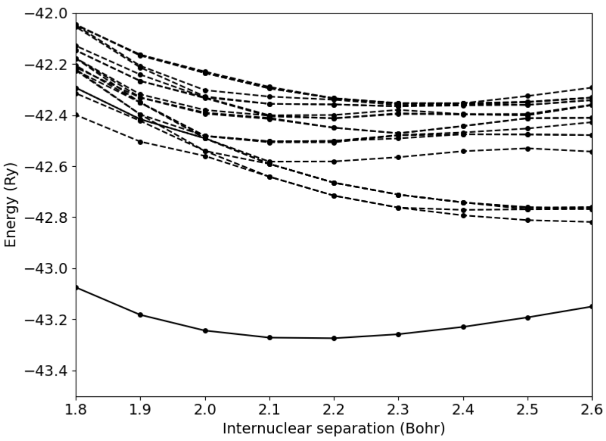

Figure S1: Total energies of the CO molecules in the ground states (bold) and in the first twenty lowest excited states (dotted) as a function of the C-O internuclear distance

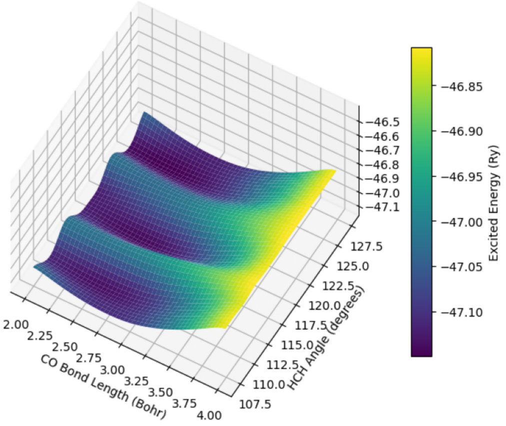

(a) C-O bond length vs HCH angle

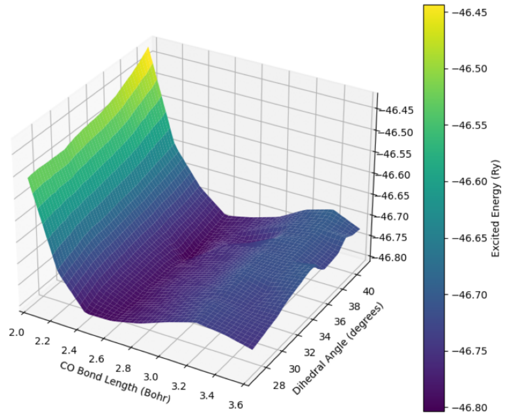

(b) C-O bond length vs Dihedral angle

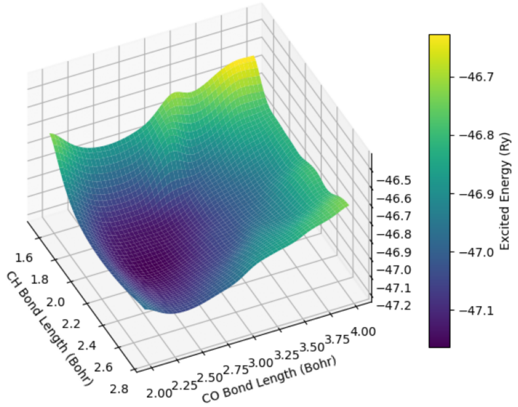

(c) C-H bond length vs C-O bond length

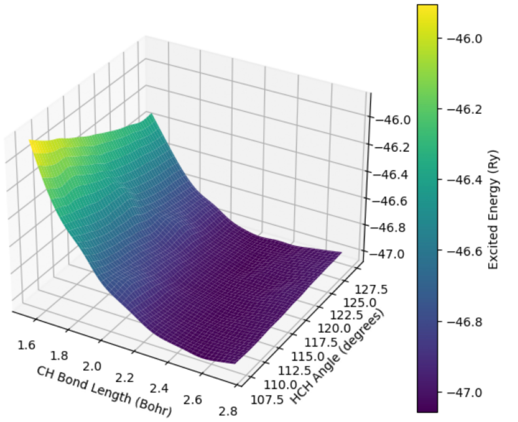

(d) C-H bond length vs HCH angle

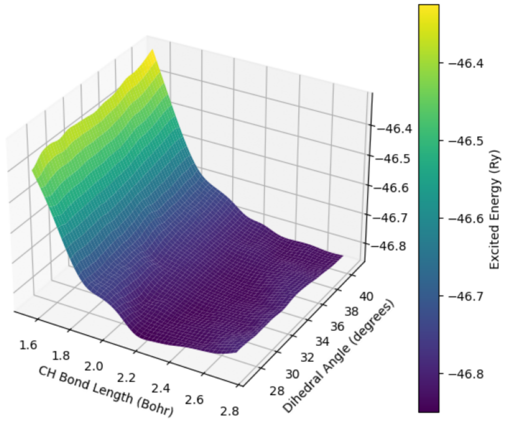

(e) C-H bond length vs Dihedral angle

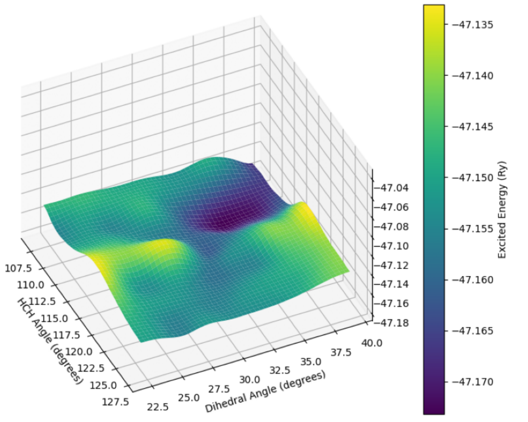

(f) HCH angle vs Dihedral angle

Figure S2: Two-dimensional scans of the first excited potential energy surface for CH_2O molecule. Each subplot [a-f] varies two internal coordinates (CO bond length, CH bond length, H–C–H angle, dihedral angle), while the other degrees of freedom are held fixed at the equilibrium values obtained from our optimization (See.Table.3). Note plot.(a) shows multiple local minima separated by small energy barrier; however, our initial guess placed the optimizer into the central minima where it will remain.
